# Fabrication and optimization of freeze-dried isoniazid-loaded poly-*ε*-caprolactone nanoparticles

**DOI:** 10.5599/admet.2774

**Published:** 2025-07-08

**Authors:** Eknath Kole, Yuvraj Pawara, Atul Chaudhari, Aniruddha Chatterjee, Jitendra Naik

**Affiliations:** 1 Department of Pharmaceutical Technology, University Institute of Chemical Technology, KBC North Maharashtra University, Jalgaon MS 425001, India; 2 R. C. Patel Institute of Pharmaceutical Education and Research, Shirpur, MS 425405, India; 3 Plastics Engineering Department, Plastindia International University, Vapi, Gujarat, India

**Keywords:** Lyophilization, nanoprecipitation, microreactor, sustained release, pulmonary tuberculosis

## Abstract

**Background:**

Microfluidic nanoprecipitation followed by freeze-drying would yield uniformly sized, stable nanoparticles by preserving their physicochemical property without compromising therapeutic performance. The isoniazid (INH)-loaded poly-*ε*-caprolactone (PCL) nanoparticles could be developed using a microfluidic technique for the management of tuberculosis.

**Experimental approach:**

The INH-loaded nanoparticles were fabricated via a microreactor-assisted nanoprecipitation method and optimization using a design of experiments factorial design approach. The resulting INH-PCL nanoformulation was characterized for particle size, polydispersity index (PDI), zeta potential (surface charge), Fourier-transform infrared spectroscopy, differential scanning calorimetry, X-ray diffraction analysis and field emission scanning electron microscope.

**Key results:**

The optimized nanoparticles exhibited an average particle size (248.4 ± 5.372 nm) and high encapsulation efficiency (82.26 ± 4.36 %). Thermal and spectroscopic analyses confirmed the absence of drug-polymer interactions, ensuring formulation integrity; stability studies under accelerated conditions demonstrated negligible changes in particle size, PDI, and zeta potential over the period of 6 months, indicating robust colloidal stability. A scanning electron microscopy study revealed rod-shaped nanoparticles with smooth surfaces. Lyophilization (freeze-drying) enhanced long-term stability, yielding a readily re-dispersible powder (reconstitution index ~1.066). Following diffusion-controlled kinetics, in vitro drug release studies in phosphate buffer saline (pH 7.4) showed sustained drug release (92.45 % cumulative release over 48 h).

**Conclusion:**

Our results confirm that the INH-loaded PCL nanoformulation combines excellent stability, high drug-loading capacity, and sustained release, key attributes of effective tuberculosis therapy.

## Introduction

Tuberculosis (TB), a life-threatening respiratory infection caused by *Mycobacterium tuberculosis* (*Mtb*), continues to pose a significant global health burden. The World Health Organization's 2024 report documented 10.8 million new TB cases and 1.25 million fatalities in 2023, underscoring the urgent need for improved treatments [1-3]. The TB infection cycle begins when aerosolized *Mtb* bacilli from an active case are inhaled into the lungs of a new host. Upon reaching the alveoli, the bacteria are phagocytosed by macrophages, ultimately leading to the development of characteristic granulomatous lesions where the host attempts to isolate the persistent mycobacteria [4]. The gradual breakdown of granuloma integrity can enable *Mtb* reactivation and spread, resulting in destructive cavitary lung lesions. Successful TB treatment must address two distinct bacterial populations: latent organisms within granulomas and actively dividing bacilli in cavities, highlighting the need for multifaceted therapeutic solutions [5]. Conventional TB treatment follows a two-phase protocol, beginning with a 2-month course of four first-line drugs (isoniazid, rifampicin, pyrazinamide, and ethambutol), followed by a 4-month maintenance phase with isoniazid and rifampicin [6]. The extended 4 to 6-month treatment course and substantial daily medication load frequently result in therapeutic challenges. These include decreased patient adherence caused by adverse effects and pharmacological interactions, which may contribute to unsuccessful treatment outcomes and the emergence of drug-resistant tuberculosis strains [7].

Several factors complicate tuberculosis treatment adherence, including the requirement for multi-drug regimens, significant treatment expenses, and undesirable medication effects. These challenges are exacerbated by the poor taste of many antitubercular formulations, creating particular difficulties for children and often resulting in skipped medications that may compromise treatment success [8,9]. Clinical studies indicate that over 10 % of tuberculosis patients undergoing conventional first-line treatment (including isoniazid therapy) terminate their regimens hastily due to medication-related complications, particularly peripheral nerve damage and liver toxicity [10,11]. Advances in pharmaceutical engineering have facilitated the design of innovative drug delivery platforms, including nanoparticle-based carriers, lipid vesicles, and polymeric microparticles [12-17]. These systems enable the controlled co-delivery of multiple therapeutic agents with prolonged release kinetics [18,19]. Engineered nanodelivery systems demonstrate superior drug-carrying capacity, programmable release kinetics, and precise tissue targeting while meeting stringent biocompatibility requirements for medical use [20,21].

The development of polymeric nanocarriers has introduced new possibilities for precision medicine. Their biodegradable composition capitalizes on physiological transport mechanisms, including vascular leakiness in diseased tissues, to enhance drug localization while sustaining therapeutic plasma levels through prolonged circulation kinetics [22-24]. Among synthetic biodegradable polymers, poly-ε-caprolactone (PCL) stands out for drug delivery applications due to its unique pharmaceutical advantages. Its demonstrated safety profile, predictable degradation behaviour, and formulation flexibility make it ideal for nanoparticle development. Researchers particularly value PCL's hydrophobic nature and permeability characteristics for sustained drug delivery. Notably, its degradation yields 6-hydroxycaproic acid, a biologically benign byproduct that preserves local pH stability [25,26].

Implementing QbD principles in nanoparticle development allows for a comprehensive evaluation of critical quality attributes. Through design of experiments (DoE) methodologies, formulators can simultaneously (i) determine essential process parameters, (ii) minimize required experimental runs, and (iii) assess multivariate factor relationships, significantly streamlining nanomedicine development [27]. This study introduced a novel approach by combining microreactor-assisted nanoprecipitation with freeze-drying to fabricate and optimize isoniazid (INH)-loaded PCL nanoparticles (NPs). This strategy leverages the precision of microreactors to achieve monodisperse NPs with high drug encapsulation efficiency (EE) while ensuring long-term stability through lyophilization critical advancement for clinical translation. This work aims to systematically develop and characterize freeze-dried INH-loaded PCL nanoparticles, optimizing key parameters like flow rate, polymer-drug ratio and surfactant concentration to enhance nanoparticle properties such as particle size, zeta potential, EE and drug release study. Microfluidic nanoprecipitation would yield uniformly sized, stable NPs, and freeze-drying will preserve their physicochemical integrity without compromising therapeutic performance. This optimized platform could pave the way for scalable, efficient TB treatment with reduced systemic side effects. Comprehensive characterization confirmed optimal nanoparticle characteristics (size <188 nm, PDI <0.3, high EE). The lead formulation achieved sustained INH release, addressing key challenges in TB treatment.

## Materials and methods

### Materials

Isoniazid (INH) was generously provided by Lupin Ltd. (Chhatrapati Sambhajinagar, India), while poly(ε-caprolactone) (PCL) and lactose were obtained from Sigma-Aldrich (USA). Poloxamer 188 (P-188) was purchased from BASF (Mumbai, India). Mannitol was obtained from Hi-Media, Mumbai. All solvents, including methanol, acetone, and glacial acetic acid, along with sodium chloride, potassium chloride, hydrochloric acid, and sodium hydroxide, were procured from Sisco Research Laboratories Pvt. Ltd. (Mumbai, India). Disodium hydrogen phosphate, potassium dihydrogen phosphate, and Tween 80 were purchased from Merck India. All chemicals and reagents used are of analytical grade purity.

### Preparation of nanoparticles

The INH-loaded PCL nanoparticles were prepared using a microreactor-assisted nanoprecipitation technique. The fabrication involved the following steps: First, 100 mg of PCL was dissolved in acetone under sonication for 15 minutes (designated organic phase-Solution A), and Tween 80 was used as a stabilizer. Simultaneously, an aqueous phase (Solution B) containing 0.1 wt.% poloxamer 188 (copolymer; surfactant) was prepared. Separately, 50 mg of INH was dissolved in 5 mL of double-distilled water and subsequently added to Solution B dropwise using a syringe. Solutions A (organic phase) and B (aqueous phase) were each stirred separately under constant magnetic agitation (600 rpm, 30 min) and then filtered through a 0.45 μm PVDF membrane (BSB Pharma, Thane) for further use. The filtered solutions were subsequently processed through a microreactor system (Amar II, Equipment, Mumbai) connected to an infusion pump (Uni Labs), maintaining a constant flow rate of 0.24 mL/min. The resulting nanosuspension was collected from the microreactor outlet under continuous stirring (400 rpm) at ambient temperature [27,28]. The INH-encapsulated nanoparticles were purified by three cycles of centrifugation (10,000 rpm, 60 min, 4 °C). As a control, blank PCL nanoparticles were prepared following the identical protocol without drug incorporation. The resuspended INH-loaded PCL nanoparticle formulation underwent pre-freezing at -20 °C for 12 h before lyophilization in a vacuum freeze-dryer (Boyikang Laboratory Instruments Inc., China) over a 48 h cycle and during the lyophilization, mannitol was added as cryoprotectant. The lyophilized nanoparticles were collected as a free-flowing powder, transferred to amber glass vials, and stored in a desiccator containing anhydrous silica gel at 25 ± 2 °C until subsequent characterization studies.

### Experimental design

The optimization of INH-loaded PCL nanoparticles or nanoformulation was investigated using a Box-Behnken experimental design, and three independent variables were evaluated at three levels [29]. Based on preliminary screening studies, two polymer concentration parameters (*X*_1_, *X*_2_) and stabilizer content (*X*_3_) were chosen as critical formulation factors. We examined three levels for the independent variables: low, medium and high, as outlined in Table 1.

**Table 1.** Experimental design framework for nanoparticle optimization: Independent variables (factors) with operational ranges and dependent variables (responses) with constraints in the Box-Behnken response surface methodology (RSM)

Input variables (independent variables)		Levels		
		Low (-1)	Medium (0)	High (+1)
Numeric factors	*X*_1_: Amount of PCL, mg	6.5	13	52
	*X*_2_: Amount of poloxamer 188, mg	5	10	20
	*X*_3_: Amount of Tween 80, % w/v (stabilizer)	0.1	0.2	0.5
Responses (dependent variables)		Constraints		
*Y*_1_: Particle size, nm		Minimum		
*Y*_2_: Polydispersity index		Minimum		
*Y*_3_: Zeta potential, mV		± less than 30		

These variables were examined for their effects on three key nanoparticle characteristics: average particle size (*Y*_1_), PDI (*Y*_2_), and surface charge (*Y*_3_). The fabricated INH-loaded PCL nanoparticles were considered concerning critical variables, including particle size, PDI, and surface charge. The experimental run, consisting of 17 randomized test conditions with triplicate runs, was generated using Design Expert^®^ software, (Stat-Ease Inc., USA, https://www.statease.com/software/design-expert/) (Table 2).

**Table 2.** Response surface methodology (RSM) outcomes for INH-loaded PCL nanoparticles: Independent variables, experimental results of dependent variables.

	Independent variables			Dependent variables		
	Factor 1 (*X*_1_)	Factor 2 (*X*_2_)	Factor 3 (*X*_3_)	Response 1 (*Y*_1_)	Response 2 (*Y*_2_)	Response 3 (*Y*_3_)
1	0	0	0	201.1	0.221	-19.3
2	0	0	0	153.2	0.235	-20.2
3	0	-1	1	267	0.086	-20.4
4	0	0	0	136.5	0.253	-16.3
5	0	1	-1	321.9	0.269	-24.3
6	-1	1	0	212.8	0.161	-21.8
7	-1	0	-1	193.1	0.295	-22.21
8	1	-1	0	169.7	0.139	-11.4
9	1	0	-1	190.3	0.209	-16.4
10	1	1	0	248.4	0.067	-24.8
11	-1	0	1	211.5	0.071	-21.3
12	0	0	0	164.9	0.119	-16.78
13	1	0	1	207.5	0.088	-14.9
14	-1	-1	0	268.4	0.105	-11.5
15	0	0	0	206.6	0.046	-17.28
16	0	-1	-1	182.23	0.261	-14.59
17	0	1	1	196.35	0.178	-15.72

The polynomial equations representing the main effects, interaction, and linear and two-factor interaction (2FI) effects of the independent formulation variables demonstrated significant impacts on the measured responses. Additionally, 3D surfaces and contour plots were used to visually illustrate the effects of the independent variables on the dependent responses. This methodology follows modern quality-focused development approaches in nanoparticle formulation, allowing efficient optimization with minimal experimental runs while ensuring reliable reproducibility. Recent nanocarrier development studies have shown that the selected experimental design has proven particularly effective for pharmaceutical process optimization [30-32].

### Particle size, polydispersity index and surface charge

The physicochemical characterization of INH-loaded PCL nanoparticles was performed using dynamic light scattering (Nano ZS 90 Zetasizer, Malvern Instruments Ltd., UK). Main parameters were measured, including hydrodynamic diameter, polydispersity index (PDI), and surface charge (zeta potential). For analysis, nanoparticle suspensions were diluted (0.1 mL nanosuspension in 2 mL deionized water) and loaded into disposable cuvettes. Measurements were conducted at 25 °C with a 173*°* backscatter angle, with each sample analysed for 120 seconds [33]. Triplicate measurements were reported as mean ± SD.

### Encapsulation efficiency and drug loading

The drug encapsulation efficiency of INH-loaded PCL nanoparticles was quantified using UV-Vis spectroscopy (UV-2600i, Shimadzu, Japan). The nanosuspension was centrifuged at 10,000 rpm for 60 min at 4 °C using a Thermo Scientific Sorvall ST 8R centrifuge to separate the free drug from the nanosuspension. The supernatant containing unentrapped drug was analysed at 262 nm, using a pre-established calibration curve (*R*^2^ = 0.9933) [27]. EE, % and drug loading (DL, wt.%) were calculated using standard formulas, Equations (1) and (2), with all measurements performed in triplicate (*n*=3).



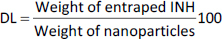

(1)




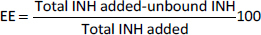

(2)


### Particle morphology

The morphological characteristics of INH-loaded PCL nanoparticles were analysed using field emission scanning electron microscopy (FE-SEM; JSM-IT300LV, JEOL, Japan). Before imaging, samples were sputter-coated with a 10 nm gold layer (JCE-3000FC coating system, JEOL, Japan) for 50 s to ensure conductivity and imaged at 10 *kV* accelerating voltage in secondary electron detection mode[31,34].

### Redispersibility

The reconstitution properties of lyophilized INH-loaded PCL nanoparticles were evaluated by dispersing 2 mg of the freeze-dried powder in 2 mL of purified water (Milli-Q grade). The redispersed nanoparticles were subsequently characterized for particle size distribution and surface charge using the dynamic light scattering technique [27].

### Fourier transform infrared spectroscopy

Fourier-transform infrared spectroscopy (FT-IR) analysis was performed using a Spectrum Two spectrometer (PerkinElmer, USA) equipped with a universal ATR accessory (diamond/ZnSe crystal). Samples were analysed in their pure form by direct placement on the ATR crystal. Spectral data were collected in transmission mode with a 4 cm^-1^ resolution, averaging 16 scans per measurement under continuous dry air purge [35]. The analysis covered the mid-infrared region (4000 to 400 cm^-1^) for pure INH, PCL, Poloxamer 188, and the INH-loaded nanoparticle formulation.

### Differential scanning calorimetry

The thermal behaviour of pure INH, PCL, Poloxamer 188, and the INH-loaded nanoparticle powder formulations was analysed using a differential scanning calorimeter (DSC-7020, Hitachi High-Tech Corporation, Japan). Approximately 5 mg of each sample was placed in hermetically sealed aluminium crucibles and subjected to a heating program from 30 to 700 °C at 20 °C per minute, under a constant nitrogen atmosphere. The thermograms obtained were analysed using TA7000 software (Hitachi High-Tech Corporation, Japan) [36,37].

### X-ray diffraction study

The powdered sample's crystalline properties and physical state were investigated using an X-ray diffractometer (Bruker AXS D8 Focus) equipped with a Cu Kα radiation source (*λ* = 0.15406 nm). The instrument was set to an operating voltage of 40 kV and a current of 40 mA. Samples of pure INH, PCL, Poloxamer 188, and the INH-loaded nanoparticle formulation were prepared by evenly spreading the powder into plastic sample holders and gently flattening the surface with a glass slide [38,39]. XRD scans were performed at ambient temperature, covering a 2*θ* angular range from 5*°* to 60*°* with a step size of 0.02*°* and a scanning rate of 1° min^-1^.

### *In vitro* aerosolization study

A Next Generation Impactor (NGI, Copley Scientific, UK) connected to a Rotahaler*^®^* (Cipla) assessed the aerodynamic properties of lyophilized INH-PCL nanoparticles. Exactly 50 mg of powder blended with lactose was loaded into size-3 capsules. Using a calibrated vacuum pump, the system operated at 60 L/min for 4 s. Upon actuation, particles are deposited across NGI stages (0.54-8.06 μm cutoff diameters). The gravimetric analysis determined emitted dose (ED), fine particle fraction (FPF ≤5 μm), mass median aerodynamic diameter (MMAD), and geometric standard deviation (GSD) [40]. Experiments were executed in triplicate (*n* = 3) to confirm reproducibility.

### *In vitro* drug release

Pure NH and INH-loaded PCL nanoparticle release profiles were evaluated under sink conditions using a dialysis bag method (MWCO: 12-14 kDa, Sigma-Aldrich). Samples (2 mL containing 50 mg of drug or formulation) were sealed in dialysis bags and immersed in 900 mL of phosphate-buffered saline (PBS, pH 7.4) maintained at 37.5 ± 0.5 °C with continuous agitation (50 rpm). 5 mL aliquots were withdrawn and replaced at predetermined intervals with fresh PBS to preserve sink conditions [41,42]. The collected samples were filtered (0.2 μm PVDF syringe filters) to remove particulates, and the filtrates were analysed for INH concentration via UV-Vis spectrophotometry (*λ =* 262 nm). All experiments were performed in triplicate (*n* = 3), with mean values reported.

### Stability study

The stability of INH-PCL nanoparticles was evaluated over 6 months under storage conditions at 25 °C. Formulations in sealed amber vials were monitored monthly for particle size, PDI and zeta potential changes to assess physicochemical stability[43].

### Statistical analysis

Experimental results are presented as mean *±* standard deviation (SD) from triplicate independent measurements. Statistical significance (*p<0.05, **p<0.01, ***p<0.001) was determined by one-way ANOVA using OriginPro 10.1 (OriginLab, https://www.originlab.com/).

## Results and discussion

This study focused on developing isoniazid-loaded poly(ε-caprolactone) nanoparticles for sustained drug release. The nanoparticles were thoroughly characterized, and a design of experiments methodology was employed to optimize formulation parameters, with particular emphasis on controlling particle size and maximizing drug encapsulation efficiency.

### Optimization of nanoparticles

BBD was used to determine the effect of the independent variable on the dependent variables. The concentration of polymers (PCL, Poloxamer 188) and stabilizer percentage (Tween 80) were selected as independent variables. The dependent variables were the particle size, PDI, and surface charge. The effect of an independent variable on the dependent variables was analysed by constructing polynomial equations and counterplots. The obtained polynomial Equations (3) to (5) for the responses particle size (*Y*_1_), PDI (*Y*_2_) and surface charge (*Y*_3_) are as follows:



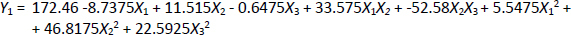

(3)




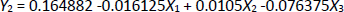

(4)




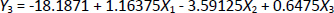

(5)


As the above equations state, the positive sign indicates the independent variable's synergic effect, whereas a negative sign indicates the antagonistic effect. The polynomial equations and statistical analysis reveal a distinct relationship between the formulation variable and nanoparticle properties. Equation (3) (particle size) exhibited synergistic and antagonistic effects of the independent variables. The negative coefficient of *X*_1_ (-8.7375) suggests an antagonistic effect, implying that increased PCL concentration reduces particle size. Conversely, *X*_2_ concentration exhibited a synergistic effect, which increased particle size, while *X*_3_ (-0.6475) had a mild antagonistic effect. A significant interaction was observed between the X_1_X_2_ (+33.575) and *X*_2_*X*_3_ (-52.58), indicating complex synergetic and antagonistic interdependencies. The optimized formulation (run 10) resulted in a minimal average particle size of 248.4±5.37 nm, highlighting the microreactor-assisted synthesis's efficiency. In Equation (4) (PDI), all three variables exhibited antagonistic or minimal effects. *X*_1_ (-0.016125) and *X*_3_ (-0.076375) contributed to a decrease in PDI, implying improved uniformity, while *X*_2_ (+0.0105) slightly increased PDI. The optimized batch (run 10) demonstrated a PDI of 0.067±0.011, indicating narrow size distributions and good nanoparticle homogeneity. Equation (5) (surface charge) showed the synergetic effect of *X*_1_ (+1.16375), increasing surface charge, whereas *X*_2_ (-3.591250 exhibited an antagonistic effect, and *X*_3_ (+0.6475) showed a mild synergetic impact. These effects reflect the electrostatic stability of nanoparticles, which is crucial for maintaining colloidal dispersion. The optimized batch (run 10) demonstrated a surface charge of -24.8 ± 0.30 mV, indicating acceptable colloidal stability of nanoparticles. The coefficient of determination (*R*^2^) values for *Y*_1_, *Y*_2_, and *Y*_3_ were 0.6633, 0.3455, and 0.3240, respectively. These values suggest a moderately good fit for the particle size model (*Y*_1_) and lower predictive accuracy for PDI (*Y*_2_) and surface charge (*Y*_3_). Nonetheless, the p-values of 0.0281 (*Y*_1_), 0.0368 (*Y*_2_), and 0.0447 (*Y*_3_) confirm the statistical significance of the models at the p < 0.05 level (Table 3).

**Table 3.** Statistical summary of the Box-Behnken design (BBD) model for optimization of INH-loaded PCL nanoparticles

	Source	Sequential *p-*value	Lack of fit *p-*value	Adjusted *R*^2^	Predicted *R*^2^
Particle size	Linear	0.8804	0.1199	-0.1712	-0.7151
	Two-factor interaction	0.0823	0.2021	0.1975	-0.4263
	Quadratic (suggested)	0.0281	0.7024	0.6633	0.1901
	Cubic (aliased)	0.7024		0.5714	
PDI	Linear (suggested)	0.0368	0.9126	0.3455	0.1786
	Two-factor interaction	0.6337	0.8827	0.2776	-0.1068
	Quadratic	0.4683	0.9532	0.2658	0.1613
	Cubic (aliased)	0.9532		-0.1916	
Zeta potential	Linear (suggested)	0.0447	0.0653	0.3240	-0.1066
	Two-factor interaction	0.1725	0.0831	0.4546	-0.5720
	Quadratic	0.9293	0.0334	0.2666	-3.5006
	Cubic (aliased)	0.0334		0.8245	

Analysis of variance (ANOVA) results in Table 4 further validated model adequacy. The interaction terms were statistically significant, particularly for *Y*_1_ (*p <* 0.0015), with an associated *F-*value of 0.4996. The *F-*values for *Y*_2_ and *Y*_3_ were 0.3489 and 5.12, respectively, indicating that the model terms contribute meaningfully to predicting response behaviour.

**Table 4.** Summary of ANOVA for *Y*_1_, *Y*_2_, and *Y*_3_ the response INH-loaded PCL nanoparticles

	Source	Degree of freedom	*F-*value	*p-*value	
Particle size (*Y*_1_)	Model	9	4.50	0.0300	Significant
	*A*: Amount of PCL	1	0.8389	0.3902	
	*B*: Amount of poloxamer 188	1	1.46	0.2666	
	*C*: Amount of stabilizer	1	0.0046	0.9478	
	*AB*	1	6.19	0.0417	
	*AC*	1	0.0005	0.9829	
	*BC*	1	15.19	0.0059	
	*A* ^2^	1	0.1780	0.6858	
	*B* ^2^	1	12.68	0.0092	
	*C* ^2^	1	2.95	0.1295	
	Lack of fit	3	0.4996	0.7024	Not significant
	Model	3	3.82	0.0368	Significant
PDI (*Y*_2_)	*A*: Amount of PCL	1	0.4797	0.5007	
	*B*: Amount of poloxamer 188	1	0.2034	0.6594	
	*C*: Amount of stabilizer	1	10.76	0.0060	
	Lack of fit	9	0.3489	0.9126	Not significant
	Model	3	3.56	0.0447	Significant
Zeta potential (*Y*_3_)	*A*: Amount of PCL	1	0.9848	0.3391	
	*B*: Amount of poloxamer 188	1	9.38	0.0091	
	*C*: Amount of stabilizer	1	0.3049	0.5902	
	Lack of Fit	9	5.12	0.0653	Not significant

**Figure 1.**
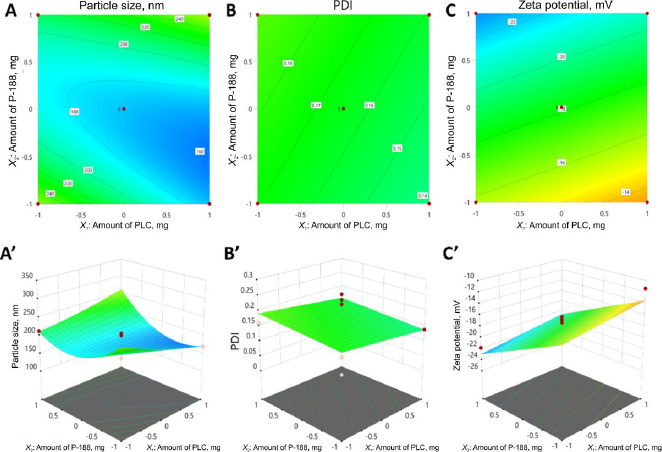
Response surface methodology (RSM) analysis of the Box-Behnken design: (A, A') 2D contour and 3D response surface plots illustrating the effect of independent variables on particle size; (B, B') polydispersity index (PDI); and (C, C') zeta potential

The 2D contour plots and 3D response surface diagrams (Figures 1A-1C') were generated to elucidate the relationships between variables.

These graphs effectively map the influence of independent variables on the characteristics of INH-loaded PCL nanoparticles, which aids in identifying optimal experimental conditions while reinforcing the statistical findings.

### Particle size, PDI and zeta potential

**Figure 2.**
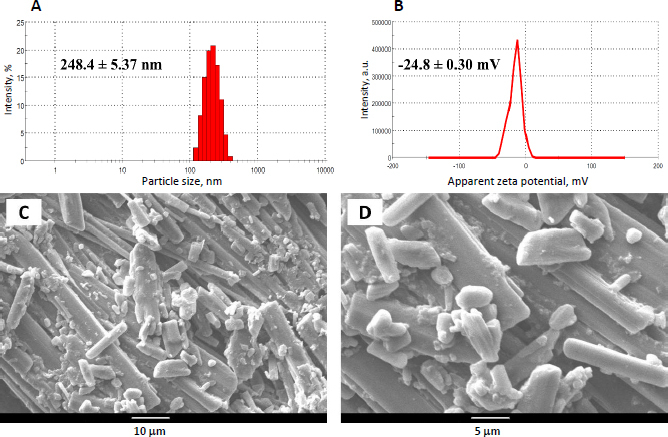
Physicochemical characterization of INH-loaded PCL nanoparticles: (A) hydrodynamic size distribution, (B) surface charge (zeta potential) (dynamic light scattering, DLS), Data represent mean ± *SD* (*n* = 3) and (C, D) scanning electron microscopy (SEM) micrographs (acceleration voltage: 10 kV)

The optimized INH-loaded PCL nanoformulation exhibited favourable physicochemical properties with a mean hydrodynamic diameter of 248.4±5.37 nm (Figure 2A), indicating successful nanoparticle formation. The narrow polydispersity index (0.067±0.011) confirmed a homogeneous size distribution, while the zeta potential of -24.8±0.30 mV (Figure 2B) suggested good colloidal stability. These results validate the effectiveness of the microreactor-assisted nanoprecipitation method for producing INH-loaded PCL nanoparticles with optimal characteristics.

### Entrapment efficiency and drug loading

The encapsulation efficiency of the lyophilized INH-loaded PCL nanoparticles was evaluated by analysing the supernatant. This involved quantifying the unencapsulated INH in the aqueous phase and comparing it to the initial theoretical drug input. The INH-loaded PCL nanoparticles exhibited high drug encapsulation efficiency (82.26±4.36 %) and a loading capacity of 9.28±0.28 wt.% (mean ± SD, *n* = 3). Several studies utilizing PCL-based nanoparticle systems have reported drug loading capacities and encapsulation efficiencies comparable to our results. Notably, Zhang *et al*. [44] research employing PCL/Poloxamer 188 nanoparticles achieved a loading capacity of 10.05 %, while plain PCL nanoparticles showed a slightly lower value of 9.98 %, with both systems demonstrating encapsulation efficiencies above 70 %. These findings corroborate our experimental outcomes and validate the effectiveness of PCL-based delivery systems for hydrophobic drug encapsulation [23,45]. These metrics confirm the successful entrapment of isoniazid within the polymeric matrix, highlighting the formulation's robust drug-loading capabilities.

### Particle morphology

The SEM images of lyophilized INH-loaded PCL nanoparticle formulation revealed a rod-like crystal structure with a smooth surface and smaller particles adhered, indicating agglomeration. Figure 2C-2D Freeze-drying led to particle clustering and possible crystallization, affecting overall nanoparticle morphology [27] .

### Redispersibility index

The redispersion behaviour of lyophilized INH-loaded PCL nanoparticles was thoroughly investigated to evaluate formulation stability and reconstitution potential. The lyophilized INH-PCL nanoparticles exhibited excellent redispersibility, reforming stable nanoparticles (264.96±10.87 nm) with low polydispersity (0.129±0.037) and good colloidal stability (-19.7 ± 0.60 mV) upon reconstitution. The RDI (~1.066) confirmed ideal reconstitution with minimal aggregation. These results demonstrate the successful preservation of nanoscale properties during lyophilization. The formulation maintains pharmaceutical suitability post-freeze-drying and storage.

### Fourier transform infrared spectroscopy

**Figure 3.**
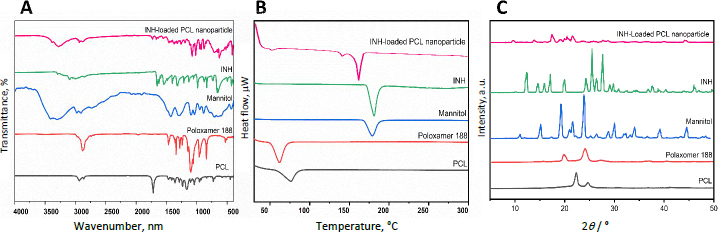
Solid-state analysis of INH-loaded PCL nanoparticles: (A) Fourier-transform infrared (FTIR) spectra, (B) differential scanning calorimetry (DSC) thermograms, and (C) X-ray diffraction (XRD) patterns

The FTIR spectrum of INH exhibited characteristic functional group peaks, including O-H stretching at 3239.82 cm^-1^, N-H stretching at 3461.69 cm^-1^, C≡N stretching at 2212.17 cm^-1^, C=O stretching at 1716.35 cm^-1^, CH_3_ stretching at 1133.34 cm^-1^, N-H bending at 1629.98 cm^-1^, pyridine ring vibrations at 1403.65 cm^-1^ and free NH_2_ at 1215.17 cm^-1^, confirming its structural integrity [46]. Similarly, PCL displayed distinct absorptions for asymmetric CH_2_ stretching (2979.00 cm^-1^), symmetric CH_2_ stretching (2893.19 cm^-1^), C=O stretching (1813.80 cm^-1^), and asymmetric COC stretching (1717.89, 1667.28 and 1149.65 cm^-1^), validating its polymer structure [47]. Poloxamer-188 showed prominent peaks for O-H stretching (3404.49 cm^-1^), aliphatic C-H stretching (2881.09 cm^-1^), in-plane O-H bending (1363.27 cm^-1^), and C-O stretching (2117.85 cm^-1^), consistent with reported data [48]. In the INH-loaded PCL nanoparticles, the spectrum revealed overlapping drug and polymer peaks with minor shifts: O-H stretching (3274.65 cm^-1^), N-H stretching (3380.81 cm^-1^), C≡N stretching (2201.84 cm^-1^), C=O stretching (1664.07 cm^-1^), CH_3_ stretching (1191.58 cm^-1^), N-H bending (1632.59 cm^-1^), pyridine ring vibrations (1430.68 cm^-1^), asymmetric CH_2_ stretching (2932.89 cm^-1^), symmetric CH_2_ stretching (2880.72 cm^-1^) and C-O/C-C stretching (1728.43 and 1664.07 cm^-1^) (Figure 3A). The absence of new peaks or significant band shifts confirms that there is no chemical interaction between INH and excipients during nanoparticle fabrication.

### Differential scanning calorimetry

Figure 3B provides valuable information on the thermal behaviour and changes in the crystallinity of INH-loaded PCL nanoparticles compared to pure INH and individual excipients. Pure INH exhibited a distinct endothermic peak at 181.36 °C, corresponding to its melting point and indicating its crystalline structure. Similarly, mannitol exhibited a sharp peak at 178.40 °C, consistent with its known crystalline nature. Poloxamer-188, functioning as a surfactant, showed a mild endothermic transition around 61.44 °C, reflecting its semi-crystalline or low-crystallinity characteristics. PCL exhibited a melting peak at 75.79 °C, typical of semi-crystalline polymers. In contrast, the DSC thermogram of the INH-loaded PCL nanoparticles revealed notable differences. The absence of the characteristic PCL melting peak indicates a disruption in the polymer's crystallinity, likely due to molecular-level interactions between INH and the PCL matrix during nanoparticle synthesis. This absence suggests that INH was successfully incorporated into the polymer network, forming an amorphous drug-polymer composite. Moreover, the melting point of INH shifted downward to 162.24 °C within the nanoparticles, suggesting a significant reduction in its crystallinity. Previously reported by Khuroo *et al*, [49] the development of rapid-dissolving printlets showed significant modification in the crystalline behaviour of INH. Their study observed the thermal behaviour of INH in the printlets formulation, which strongly supports the formation of an amorphous drug-polymer interaction or a reduction in crystallinity. This is evidenced by the shift in the INH melting peak from 174.8 °C (pure drug) to 168.3 to 168.9 °C (printlets), indicating disruption of the crystal lattice due to polymer interactions. This shift could be attributed to possible drug-polymer interactions or the formation of smaller and less stable crystalline domains of INH [49].

Additionally, a minor endothermic peak at 142.11 °C was observed, which may represent trace amounts of residual crystalline drug or interactions with excipients. However, the low intensity of this peak suggests a minimal impact on the overall system. Collectively, the DSC results demonstrate that INH was efficiently encapsulated within the PCL matrix, with notable changes in crystallinity supporting the formation of a well-integrated nanocarrier system optimized for drug delivery.

### X-ray diffraction analysis

Figure 3C shows the XRD pattern of pure INH, PCL, Polaxomer-188 and INH-loaded PCL nanoparticles across the 2*θ* range of 5 to 60˚. The XRD analysis revealed a distinct crystallinity pattern for each excipient: PCL showed characteristic peaks at 22.33 and 24.65*°*, poloxamer 188 at 19.51 and 23.68*°*, and mannitol exhibited multiple sharp peaks between 11.04 and 44.52*°*, while pure INH demonstrated intense crystalline peaks from 12.37 to 46.08*°*. The INH-loaded PCL nanoparticles displayed a modified diffraction pattern with peaks at 9.88, 17.60, 20.52 and 21.55*°*, where the absence of Sharpe peaks and reduced PCL crystallinity indicates successful INH encapsulation and partial amorphization within the polymeric matrix. These structural changes, evidenced by peak broadening and shift, correlate with the formulation's sustained release behaviour of INH while maintaining sufficient nanoparticle stability through preserved PCL crystallinity. The XRD data collectively confirm the successful fabrication of polymeric nanoparticles for drug delivery with optimal physicochemical properties for sustained or controlled release.

### *In-vitro* aerosolization properties

**Figure 4.**
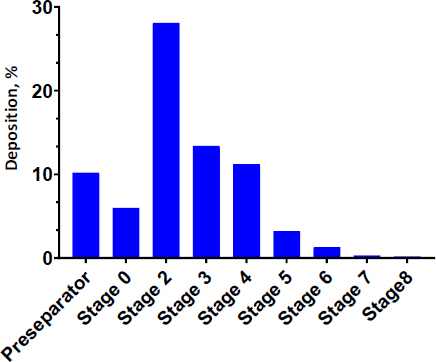
In vitro aerosolization behaviour of INH-loaded PCL nanoparticles

The aerosolization performance of INH-loaded PCL nanoparticles was evaluated using the NGI apparatus. The results of the deposition pattern demonstrated optimal particle distribution, with most drug depositions occurring below stage 3 (effective cutoff diameter *<*4.7 μm), indicating successful targeting of the bronchioalveolar region where mycobacteria reside. Our developed formulation exhibited favourable aerodynamic characteristics, including a mass medium aerodynamic diameter (MMAD) of 4.9 μm, which falls within the ideal 1-5 μm range for efficient pulmonary delivery. Notably, the fine particle fractions (PFP) reached 40.1 %, confirming that a substantial proportion of particles were within the respirable size ranges (<5 μm). The high emitted dose of 92.4 % further demonstrated excellent powder dispersion from the delivery device. The narrow GSD (1.58) suggests consistent particle size distribution, while the MMAD and FPF demonstrate compatibility with deep lung delivery. The deposition profile, illustrated in Figure 4, provides compressive evidence of the formulation's suitability for inhalation therapy. The collective findings validate that the INH-loaded PCL nanoparticle formulations possess the necessary aerodynamic properties for effective deep lung depositions, making them promising candidates for targeted therapy against pulmonary tuberculosis.

### *In-vitro* drug release study and kinetic study

**Figure 5.**
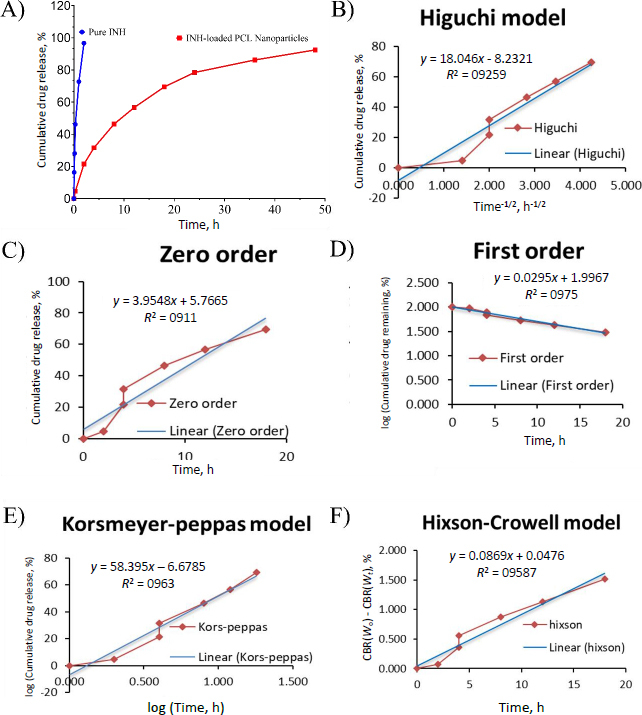
*In vitro* drug release and kinetic study. (A) cumulative release of pure INH and INH-loaded PCL nanoparticles (*n* = 3; mean ± SD). Release kinetics fitted to (B) Higuchi, (C) zero-order, (D) first-order, (E) Korsmeyer-Peppas and (F) Hixson-Crowell models. (CBR - cumulative burst release, *W*_o_ - initial weight, and *W_t_* - weight at time)

The *in vitro* drug release profiles of pure INH and INH-loaded PCL nanoparticles were evaluated using a dialysis method in PBS (pH 7.4). Pure INH exhibited a rapid release, with 96.66 % of the drug released within the first 2 h, indicating an immediate-release profile. In contrast, the INH-loaded PCL nanoformulation showed a sustained release pattern, with 21.50 % released at 2 h, 78.43 % at 24 h, and 92.45 % over 48 h. This extended-release behaviour highlights the controlled release capability of the PCL nanoparticle matrix, which can help maintain therapeutic drug levels over a prolonged period. Such a release profile offers significant therapeutic advantages, including reduced dosing frequency and improved patient compliance compared to conventional formulations. (Figure 5A) The drug release behaviour of INH-loaded PCL nanoparticles was analysed using various kinetic models, including zero-order, first-order, Higuchi, Hixson-Crowell, and Korsmeyer-Peppas models. (Figures 5B-5F) Among these, the first-order model exhibited the highest correlation coefficient (*R*^2^ *=* 0.9750), indicating that the release rate depends on the remaining drug concentration and follows a concentration-gradient-driven mechanism. The zero-order (*R*^2^ *=* 0.911) and Hixson-Crowell (*R*^2^ *=* 0.9587) models also showed good fits, suggesting a controlled release profile possibly influenced by surface area reduction and matrix erosion. The Higuchi model (*R*^2^ *=* 0.9259) revealed diffusion-based release through the polymer matrix (Table 5). The kinetic constant (*k*) was determined to be 3.9548, while the release exponent (*n*) of the Korsmeyer-Peppas equation was found to be 6.6785. The high value of the release exponents (*n >*0.89) indicates that Super Case II transport (non-Fickian diffusion) governs the drug release. These findings strongly suggest that the dominant release mechanism involves substantial polymer relaxation and matrix swelling effects, rather than simple diffusion-controlled release [45].

**Table 5.** Release kinetic models of INH-loaded PCL nanoparticles

Model	INH-loaded PCL nanoparticles		
	*R* ^2^	*k*	*n*
Zero order	0.9110	3.9548	5.7665
First order	0.9750	0.0295	1.9967
Higuchi model	0.9259	18.046	8.2321
Hixson-Crowell model	0.9587	0.0869	0.0476
Korsmeyer-peppas model	0.9630	58.395	6.6785

Overall, the findings indicate that INH release from the nanoparticles is primarily governed by first-order kinetics, with additional contributions from diffusion and polymer degradation processes, supporting its potential for sustained drug delivery.

### Stability study

**Figure 6.**
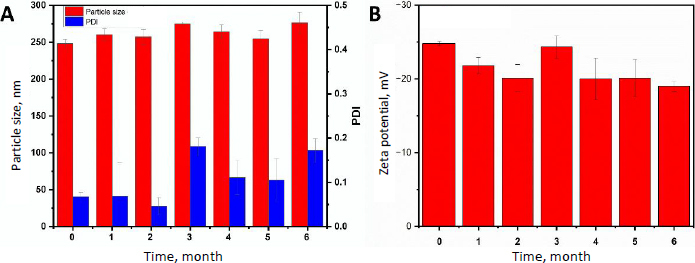
Stability evaluation of INH-loaded PCL nanoparticles stored under controlled conditions for six months: (A) changes in particle size and polydispersity index and (B) zeta potential (mean±SD, *n*=3).

The long-term stability of lyophilized INH-loaded PCL nanoparticles was evaluated under controlled storage conditions (25±2 °C) for six months. Throughout the study period, key physicochemical properties, such as particle size, zeta potential, and PDI, remained within acceptable ranges (p > 0.05), indicating no significant variations. The formulation also maintained its structural integrity, with no visible changes observed in its appearance. These findings demonstrate that the nanoformulation remains stable under ambient storage conditions (Figure 6A and B).

## Conclusions

In this study, we successfully developed freeze-dried isoniazid (INH)-loaded PCL nanoparticles using a microreactor-assisted nanoprecipitation method optimized through a BBD. The optimized formulation exhibited a uniform particle size of 248.4±5.372 nm, high drug encapsulation efficiency (82.26±4.36 %), and excellent colloidal stability under accelerated storage conditions over six months. Comprehensive physicochemical characterization (FTIR, DSC, XRD) confirmed the absence of drug-polymer interactions, while SEM analysis revealed a rod-like crystal structure and smooth-surfaced nanoparticles. In vitro release studies showed sustained, diffusion-controlled drug release, with 92.45 % cumulative release over 48 h. This INH-loaded PCL nanoformulation effectively addresses key challenges in tuberculosis therapy by combining high drug-loading capacity, prolonged release, and robust stability, offering a promising strategy to reduce dosing frequency and enhance patient compliance in pulmonary TB treatment. Future studies will focus on in vivo pharmacokinetic and biodistribution assessments to further evaluate the formulation's therapeutic potential and lung-targeting efficiency.
